# Healthcare professionals’ views on training, standards, and resources for extracorporeal membrane oxygenation: a cross-sectional survey

**DOI:** 10.3325/cmj.2025.66.419

**Published:** 2025-12

**Authors:** Bekzhan A. Permenov, Olena Zimba, Marlen Yessirkepov, Darkhan Suigenbayev, Burhan Fatih Kocyigit

## Abstract

**Aim:**

To assess health care professionals’ knowledge and opinions regarding extracorporeal membrane oxygenation (ECMO) use, training, standards, and resource availability.

**Methods:**

This cross-sectional study employed an online self-administered survey to evaluate health care professionals' knowledge and opinions concerning ECMO procedures. The survey consisted of multiple-choice and open-ended questions inquiring about demographics, ECMO practices, training and certification experiences, ECMO use during the COVID-19 pandemic, and obstacles to ECMO implementation.

**Results:**

The study enrolled 89 health care professionals from 12 countries. The respondents were most frequently from Kazakhstan (67.4%), Turkey (5.6%), Croatia (5.6%), and Ukraine (5.6%). Notably, 61.8% of respondents supported ECMO procedures performed by certified specialists. The respondents believed that the main ECMO indications were respiratory failure (83.1%), cardiopulmonary failure (69.6%), heart and lung transplantation (64.1%), and cardiogenic shock (58.4%). Major obstacles to ECMO implementation were reported to be high costs (53.9%), inadequately qualified staff (52.8% for physicians, 41.6% for nurses), and restricted availability of ECMO devices (42.7%).

**Conclusion:**

The findings emphasize the need for standardized training, wider availability of ECMO standards, and efforts to address resource-related barriers to ECMO access. Our results primarily reflect practices in Kazakhstan and should be interpreted in light of the study's restricted geographical coverage.

Extracorporeal membrane oxygenation (ECMO) is a complex life-sustaining procedure intended for individuals with refractory cardiac or respiratory failure unresponsive to conventional procedures (1). ECMO enhances gas exchange and hemodynamic stability by channeling the patient's blood through an external oxygenator, functioning as an artificial lung, and can either assist or completely substitute cardiac function (2). This advanced technology has been widely utilized in critical care, particularly for acute respiratory distress syndrome (ARDS), cardiac shock, and perioperative assistance during transplantation (3-5). In the COVID-19 pandemic, ECMO had been prioritized as a crucial therapeutic alternative for patients experiencing severe acute respiratory failure non-responsive to mechanical ventilation, functioning as a life-saving measure in severe viral pneumonia and respiratory failure (6,7).

The increasing use of ECMO has generated considerable interest in its clinical effectiveness and safety. Survey-based research is essential since it provides real-world data on practice trends, clinical experiences, and health care professionals' views on ECMO procedures (8-10). Such studies are used to identify knowledge gaps, practice disparities, and obstacles to the broad adoption of ECMO, particularly in resource-limited health care systems. Furthermore, survey approaches enable evaluation of ECMO training programs, underscoring their efficacy in skill development and the essential contribution of multidisciplinary teams to improved patient survival and procedural outcomes (11,12).

Despite the growing body of clinical data supporting ECMO use, there is limited information on how health care professionals perceive ECMO training, certification standards, and institutional readiness, particularly in resource-limited and transitional health care settings. Rather than broadly assessing all aspects of ECMO practice, the current study focuses on health care professionals' perspectives on ECMO training, certification standards, and resource-related barriers, which are the most critical determinants of safe and sustainable ECMO implementation.

The aim of the survey was to explore the health care professionals’ views on ECMO availability, prominent indications, the perceived efficacy and safety of the intervention, and obstacles to ECMO implementation. These domains reflect the essential structural, clinical, and organizational aspects that directly impact decision-making, patient selection, and the practicality of ECMO use in routine clinical settings. Assessing availability and indications helps identify disparities in access and clinical prioritization, whereas evaluating perceived efficacy, safety, and implementation barriers sheds light on professional confidence, institutional readiness, and system-level constraints affecting ECMO delivery.

## Respondents and methods

This cross-sectional study used an online self-administered survey to assess health care professionals' knowledge, perceptions, and practices concerning ECMO procedures. The target population comprised health care professionals engaged in the care of critically ill patients, including physicians, nurses, and allied health care professionals from diverse specialties. The survey employed the *SurveyMonkey.com* platform.

### Survey design

The questionnaire items were formulated based on an extensive review of relevant and evidence-based literature. The questionnaire primarily adhered to the guidelines of the Extracorporeal Life Support Organisation (ELSO) (https://www.elso.org/ecmo-resources/elso-ecmo-guidelines.aspx).

The questionnaire addressed ECMO definitions, indications, functions, components of the ECMO team, types of ECMO, and complications. It encompassed aspects of ECMO use during crises such as the COVID-19 pandemic. It also addressed the qualifications of ECMO team members and the obstacles they faced. Furthermore, it inquired about ECMO-related training protocols.

Five experts reviewed the survey content to improve its clarity and ensure consistency and validity. Two stages of revisions were conducted. In the pretest stage, ten health care professionals from various disciplines were contacted to complete the survey on the *SurveyMonkey.com* platform. The findings were evaluated, and the questionnaire was modified according to the received feedback.

The final version of the questionnaire (Supplemental Material 1(Supplementary material 1)) is divided into four sections: ECMO-related definitions, knowledge, and experiences; ECMO-related training; barriers to ECMO use and ECMO in the pandemic period; and sociodemographic information. The questionnaire consists of 37 questions: 22 multiple-choice, 7 Likert-type (on a 1-5 scale, with 1 – not important and 5 – extremely important), and 1 open-ended question. The completion time was approximately 10 minutes.

Respondents were permitted to modify their responses before concluding the survey but not afterwards. To guarantee that incomplete responses were promptly eliminated, all inquiries were designated as mandatory on the SurveyMonkey platform.

### Sampling

Between July 23, 2024 and September 26, 2024, the questionnaire link was disseminated on X (Twitter), Facebook, and WhatsApp. The study investigators distributed the survey link throughout the study period to engage a convenience sample of health care professionals. No specific sampling approach was employed; participation was voluntary and self-selected.

The study was approved by the Ethics Committee of the South Kazakhstan Medical Academy. Before completing the survey, respondents were informed that their answers would only be used for research purposes, and their informed consent was obtained.

### Confidentiality, integrity, and availability

The research employed internet protocol (IP) identifiers and respondent emails as the sole identifying indicators of the respondents. The moderator ensured data confidentiality by securing IP addresses and emails. The synthesized data were subsequently accessible in anonymized databases. This report adheres to the principles of designing and reporting survey studies (13).

### Statistical analysis

Descriptive data are presented as numbers and percentages. The differences between the groups were evaluated with the χ^2^ test. The level of statistical significance was set at *P* < 0.05. The statistical analysis was conducted with SPSS, version 25.0 (IBM Corp., Armonk, NY, USA).

## Results

### Respondents’ characteristics

**Figure 1:**
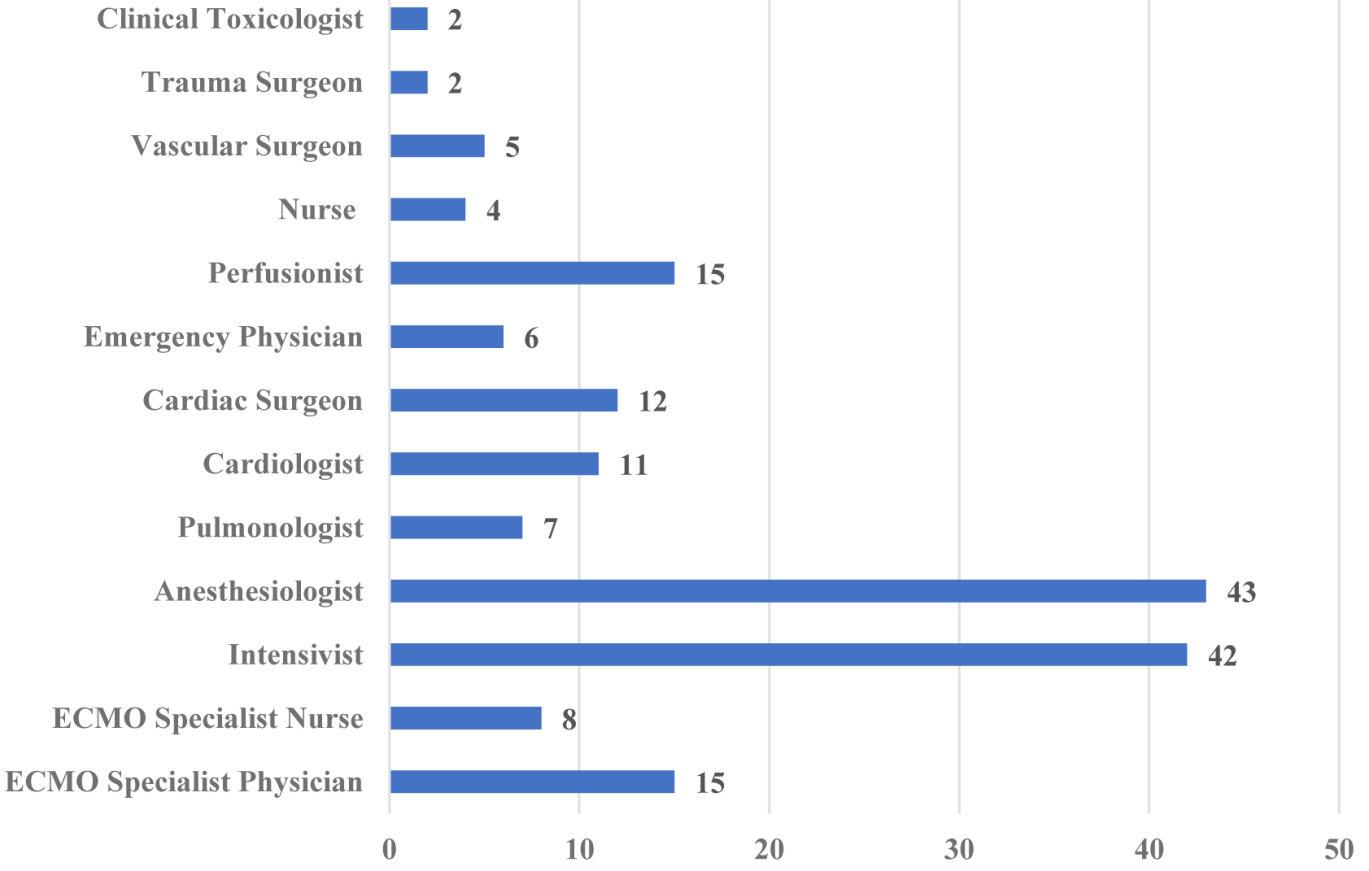
Respondents' specialties. ECMO – extracorporeal membrane oxygenation.

The survey was completed by 89 individuals, with a median age of 40 (range 24-84) years. Most of the respondents were anesthesiologists (n = 43, 48.3%) and intensivists (42 or 47.2%) (Figure 1). Respondents worked in public hospitals (n = 64, 71.9%), university-affiliated hospitals (n = 14, 15.7%), private hospitals (n = 9, 10.1%), and other institutions (n = 2, 2.3%). Thirty (33.7%) participants indicated that their health care facilities possessed a specialized department or unit for ECMO. Eighty-three (93.3%) worked in urban health care settings, whereas 6 (6.7%) worked in rural areas.

Among the participants, 11 (12.4%) had been practicing as health professionals for less than 1 year, 21 (23.6%) for 1 to 5 years, and 57 (64%) for more than 5 years. The participants' experience in emergency medicine was as follows: 10 (11.2%) participants had less than 1 year of experience, 21 (23.6%) had 1 to 5 years of experience, and 58 (65.2%) had more than 5 years of experience.

**Figure 2:**
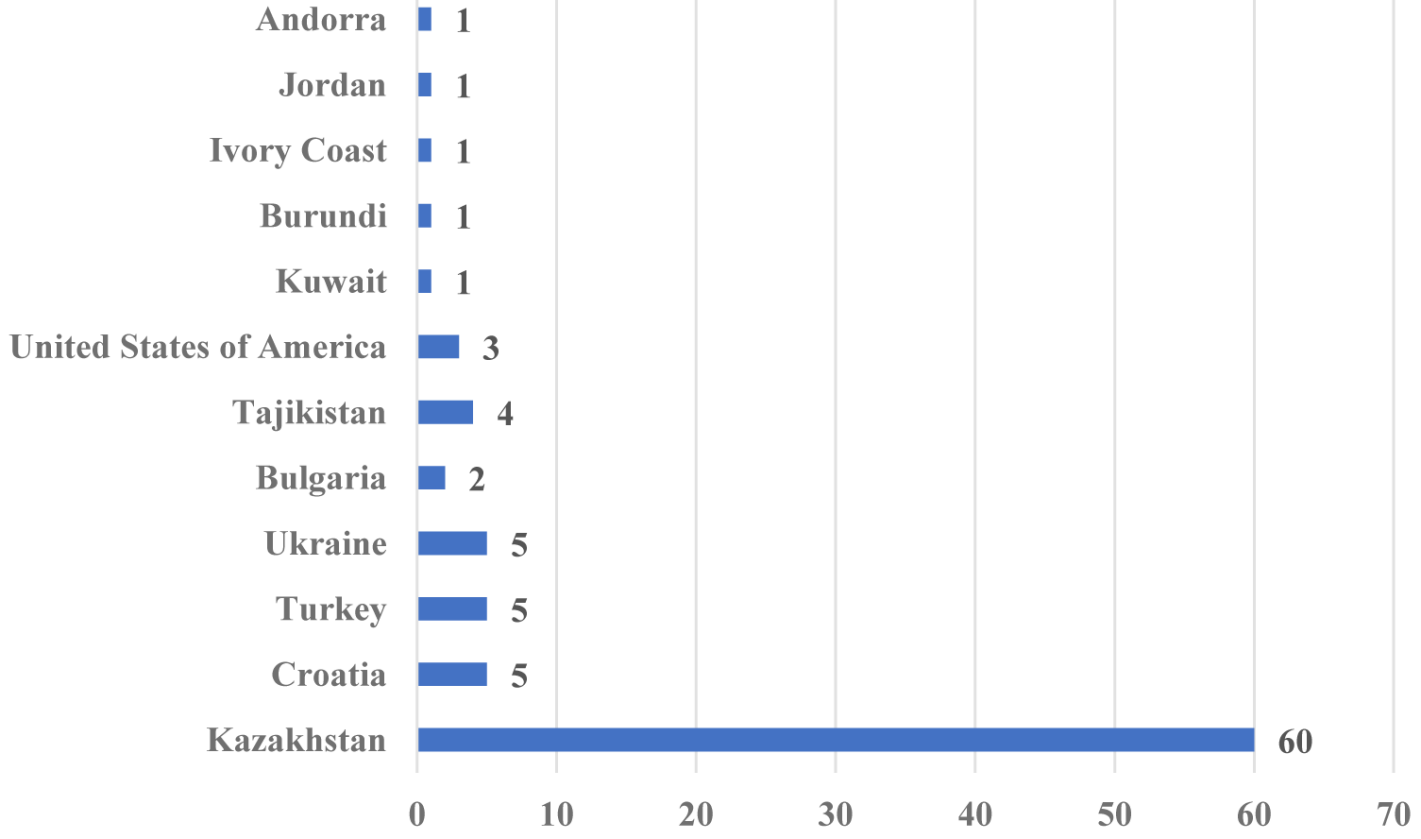
Respondents’ countries of origin.

The respondents originated from 12 countries, mostly Kazakhstan (n = 60, 67.4%), Turkey (n = 5, 5.6%), Croatia (n = 5, 5.6%), and Ukraine (n = 5, 5.6%) (Figure 2). In statistical analysis, countries were categorized into Kazakhstan and others. The two groups did not significantly differ in ECMO availability (*P* = 0.711, χ^2^ test), health care professional experience (*P* = 0.524), or the duration of time the participants engaged in emergency medicine (*P* = 0.199).

### Definitions, knowledge, and experiences

A total of 73 (82%) participants were familiar with the ECMO definition, and 55 (61.8%) were familiar with the Extracorporeal Life Support Organization (ELSO) guidelines. Most participants agreed that ECMO was effective and safe for critically ill patients (60.7%), while 27.0% were neutral and 12.4% disagreed or strongly disagreed.

**Figure 3:**
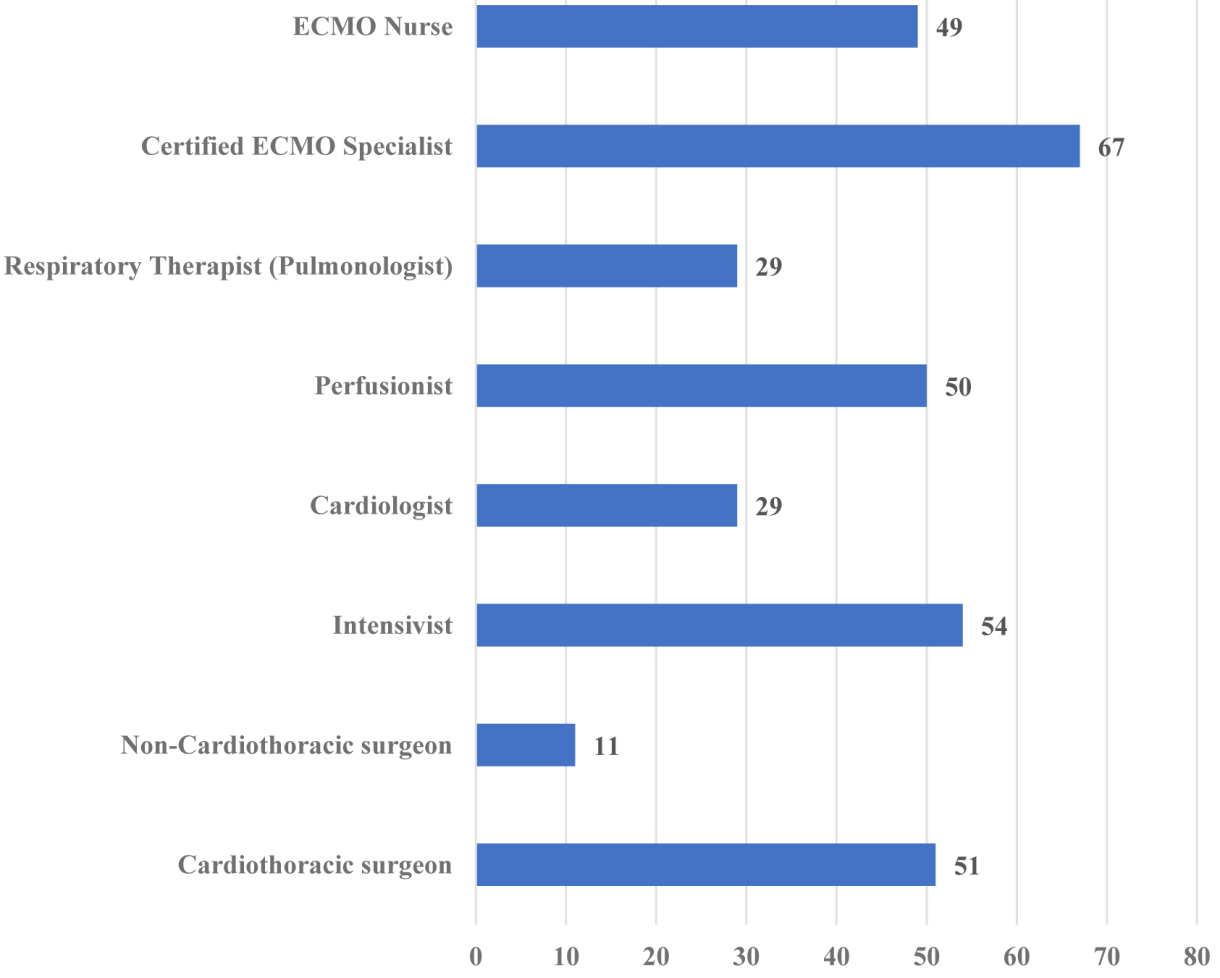
Respondents’ choice of health care professionals who should be involved in extracorporeal membrane oxygenation (ECMO) procedure to reduce risks and improve patient survival.

**Figure 4:**
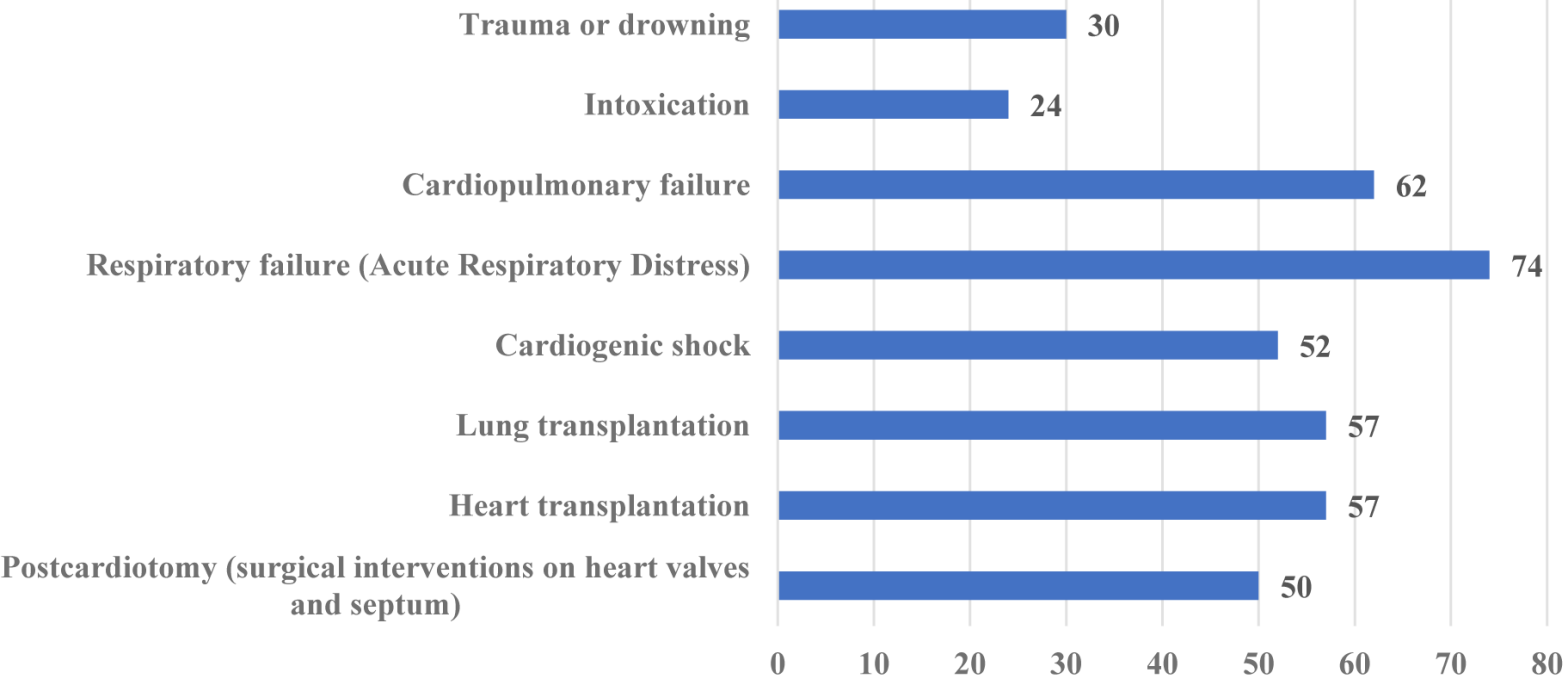
Extracorporeal membrane oxygenation (ECMO) indications as reported by the respondents.

**Figure 5:**
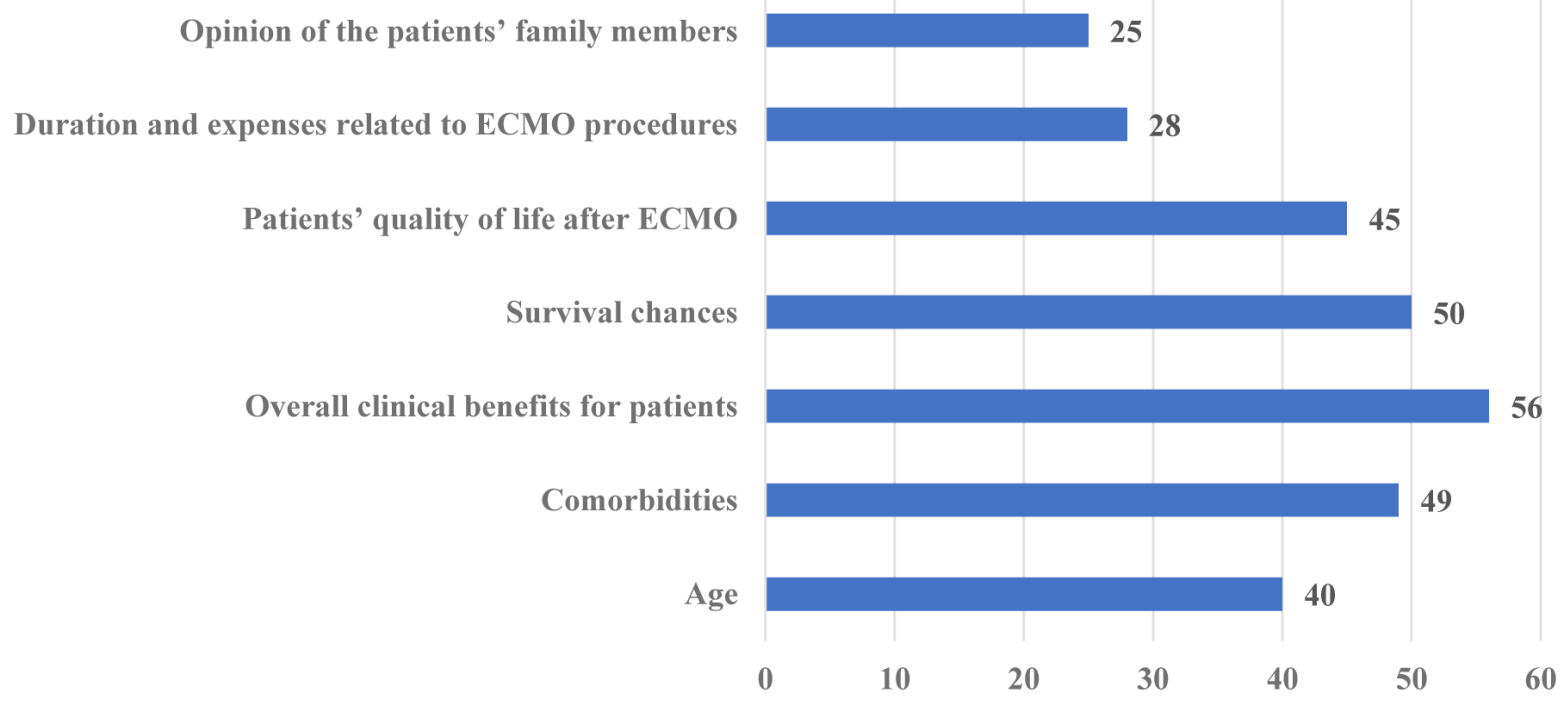
Factors considered when deciding to initiate or discontinue extracorporeal membrane oxygenation (ECMO) as reported by the respondents.

Respondents believed that health care professionals who should be involved in ECMO procedures were primarily certified ECMO specialists (75.3%), intensivists (60.7%), cardiothoracic surgeons (57.3%), perfusionists (56.2%), and ECMO nurses (55.1%) (Figure 3). Most respondents agreed that ECMO should be administered only by certified specialists (61.8%), while 12.4% were neutral and 25.8% disagreed or strongly disagreed. Respondents most frequently reported respiratory failure (n = 74, 83.1%), cardiopulmonary failure (n = 62, 69.6%), and heart and lung transplantation (n = 57, 64.1%) as indications for ECMO (Figure 4). The factors most commonly considered when initiating or discontinuing ECMO included overall clinical benefit (n = 56, 62.9%), survival chances (n = 50, 56.2%), comorbidities (n = 49, 55.1%), and predicted post-ECMO quality of life (n = 45, 50.6%) (Figure 5).

**Figure 6:**
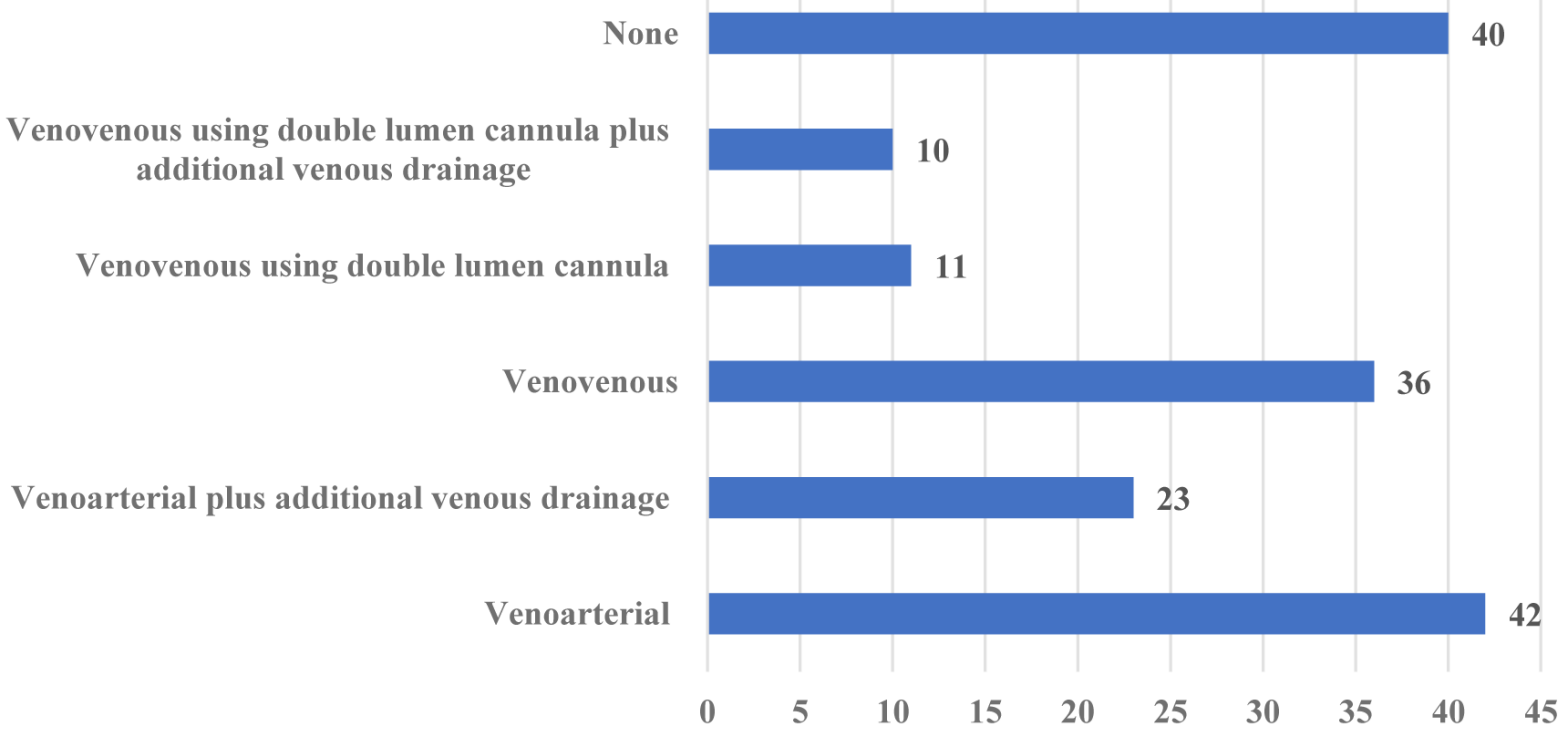
The extracorporeal membrane oxygenation (ECMO) modes offered in medical centers as reported by the respondents.

Overall, 48 participants (53.9%) reported that their medical centers offered ECMO support. Regarding annual ECMO case volume, 34 respondents (38.2%) reported no procedures, while 37 (41.6%) reported fewer than 30 procedures per year. Concerning institutional experience, 31 participants (34.9%) reported providing ECMO for 5 years or less, 14 (15.7%) for more than 10 years, and 34 (38.2%) were not sure. The most commonly reported ECMO modes were venoarterial (n = 42, 47.2%) and venovenous (n = 36, 40.4%) (Figure 6). Anticoagulant preferences during ECMO were as follows: heparin (n = 75, 84.3%), unfractionated heparin (n = 16, 18%), argatroban (n = 5, 5.6%), bivalirudin (n = 4, 4.5%), warfarin (n = 2, 2.2%), and rivaroxaban (n = 1, 1.1%). The primary antimicrobial therapeutic strategies were based on antimicrobial characteristics (n = 59, 66.3%) and did not differ from those used in therapies for critically ill patients (n = 25, 28.1%).

### ECMO training

Thirty-one participants (34.8%) reported that ECMO training was available at their medical centers. The most frequently reported training requirements included didactic courses at a specialist center with lectures, seminars, and handouts (n = 54, 60.7%), scenario-based simulation training (n = 53, 59.6%), and residency certification in a relevant specialty (n = 45, 50.6%). Most participants rated didactic teaching by highly skilled ECMO specialists as important or extremely important; 13 respondents (14.6%) rated it as important, and 50 (56.2%) as extremely important.

Simulation-based training was rated as important or extremely important by 64 respondents (71.9%), membership in ECMO-related associations by 54 (60.6%), and research in the field of ECMO by 57 respondents (64.1%). Thirty-six participants (40.4%) reported prior authorship of ECMO-related research.

### Barriers to ECMO use and the COVID-19 period

**Figure 7:**
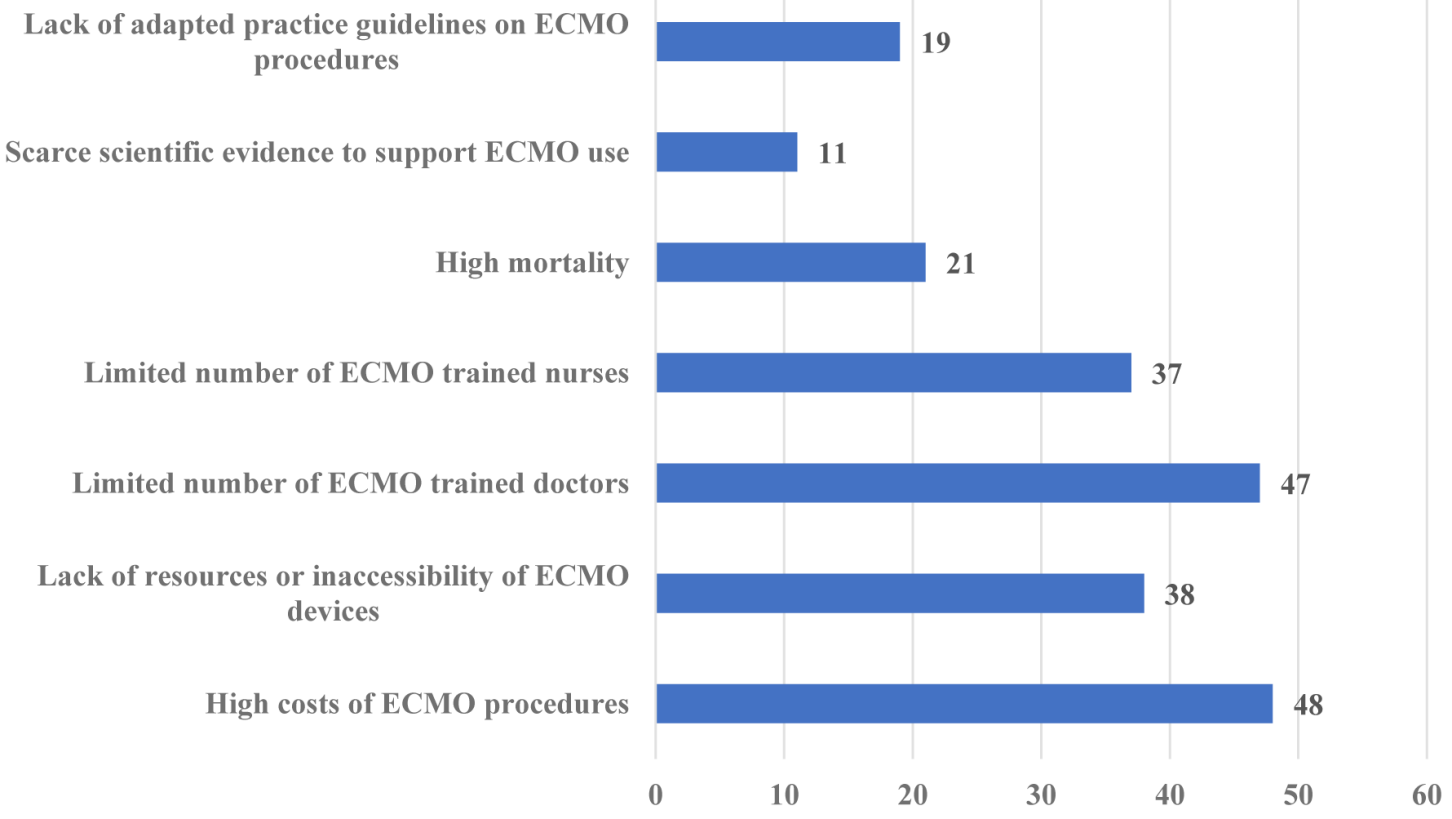
Barriers to the widespread use of extracorporeal membrane oxygenation (ECMO) as reported by the respondents.

The most frequently reported barriers to ECMO use were high procedural costs (n = 48, 53.9%) and a limited number of ECMO-trained physicians (n = 47, 52.8%) (Figure 7). Sixty (67.4%) participants managed critically ill COVID-19 patients referred to ECMO. Thirty respondents (33.7%) reported that the COVID-19 pandemic did not affect the organization of ECMO procedures in the health care setting at all, 34 (38.2%) reported that the pandemic affected it to some extent, and 25 (28.1%) that it affected it considerably. During the COVID-19 pandemic, 53 (59.6%) responders reported an increase in referrals of COVID-19 patients to ECMO, while 25 (28.1%) noted an increase in referrals of non-COVID-19 patients to ECMO. Regarding ECMO effectiveness, 56.2% of respondents agreed or strongly agreed with its use in critically ill COVID-19 patients, and 55.1% agreed or strongly agreed with its use in critically ill non-COVID-19 patients.

To improve the efficiency of ECMO procedures, 29 responders recommended ECMO training (32.6%), while other recommendations were multidisciplinary training programs, sharing of experiences, online meetings, simulation-based training, and ECMO certification programs.

## Discussion

This study suggests that, while the majority of health care professionals recognize the clinical importance of ECMO, significant deficiencies remain in training, certification, and institutional readiness to implement it. These data suggest that deficits in knowledge, workforce capability, and resources are the primary barriers to the safe and sustainable use of ECMO in current practice.

The research involved participants from 12 countries, mostly intensivists, anesthesiologists, perfusionists, and cardiothoracic surgeons. Approximately two-thirds of the responders originated from Kazakhstan. Notwithstanding the variety of participants' specialties, 71.9% worked in public institutions, suggesting that ECMO is primarily incorporated into public health care systems. Importantly, only 33.7% of respondents reported the existence of a specialized ECMO department in their health institution, which underscores the restricted availability of ECMO, particularly in resource-constrained environments (14,15). Conversely, survey-based studies in well-resourced health care systems report higher access to specialized ECMO centers, increased case volumes, and more organized training and certification options for ECMO providers (11,12). These disparities likely reflect discrepancies in health care funding, institutional structure, and the availability of qualified experts. Such variations may partially elucidate the disparities in ECMO availability, education options, and perceived obstacles reported by participants in the current study.

The majority of participants were familiar with the globally acceptable definition of ECMO, and 61.8% were familiar with relevant ELSO guidelines. The obtained results point to good understanding of ECMO among health care professionals, yet a considerable percentage still remain unfamiliar with the guidelines. This underscores the need for enhanced dissemination of ECMO-specific protocols to guarantee uniformity in practice across hospital environments (16,17).

Overall, 60.7% of respondents agreed or strongly agreed that ECMO was efficacious and safe for managing critically ill patients. A substantial proportion of respondents (37.1%) were neutral or disagreed, which might indicate uncertainty or skepticism about ECMO results, especially in high-risk patient scenarios. These findings highlight the continuing discussion on the specific indications and long-term benefits associated with ECMO, particularly in patients with multiple comorbidities or elderly individuals (18,19).

The survey responses underscored the significance of a multidisciplinary approach to ECMO, identifying cardiothoracic surgeons, intensivists, perfusionists, and certified ECMO specialists as crucial team members. Notably, 61.8% of respondents endorsed the necessity for specialists with ECMO certification to perform ECMO procedures, highlighting the importance of standardized training and formal certification. This is essential for minimizing complications and enhancing patient outcomes, especially in high-risk ECMO scenarios (20-22).

Respondents found predominant indications for ECMO to be respiratory failure, cardiopulmonary failure, heart and lung transplantation, and cardiogenic shock. These results correspond with the recognized indications for ECMO use in critically ill patients (23). Notably, trauma and intoxication were cited by 33.7% and 26.9% of respondents, respectively, indicating an increasing acknowledgment of ECMO use in non-cardiopulmonary emergencies. The expansion of ECMO indications aligns with accumulating evidence that supports its use in these situations, especially when standard treatments are ineffective (24,25).

The survey findings on anticoagulant preferences during ECMO point to heparin as a preferred drug. Heparin continues to be the standard anticoagulant in ECMO processes, perhaps owing to its efficacy and availability of related protocols.

Regarding antimicrobial treatment during ECMO, 66.3% of participants formulated their strategy based on the medications' pharmacokinetics, including protein binding qualities, molecular weight, and solubility (hydrophilic/lipophilic features). This demonstrates a focused strategy for antibacterial treatment that considers changed pharmacodynamics during ECMO.

The respondents described the main barriers to ECMO utilization as high costs, restricted availability of ECMO equipment, and a deficiency of ECMO-trained personnel. These results are in line with published reports, emphasizing expensive resource requirements, including equipment and specialized personnel (26). The current survey results highlight the need for advanced training programs to broaden the team of health care professionals qualified to properly deliver ECMO.

During the COVID-19 pandemic, ECMO use was prioritized, with 59.6% of respondents indicating increased referrals of COVID-19 patients to ECMO. This is in line with global trends since ECMO was used as a last-resort treatment for COVID-19 patients with severe ARDS resistant to mechanical ventilation (27). Opinions on the efficacy of ECMO in treating COVID-19 varied, with just 19.2% of respondents expressing significant agreement with its success in such instances. This presumably indicates the difficulties encountered during the pandemic, including patient selection, resource distribution, and elevated mortality rates regardless of ECMO assistance (28).

An important issue in the current study was the need for advanced ECMO training. Only 34.8% of participants stated that their centers offered official ECMO training, with the majority underscoring the value of didactic and simulation-based instructions. This highlights the need for organized ECMO educational programs, including practical training and certification, to provide health professionals with competencies essential for safe and successful ECMO management (29). The respondents suggested extending ECMO training programs through multidisciplinary simulation-based training and ECMO certification. These results are consistent with the survey's overall findings, emphasizing the need for a standardized ECMO education.

A limitation of the current study is a relatively small sample size and convenience sampling, which restrict the generalizability of the findings. Additionally, the respondents were predominantly from Kazakhstan. Consequently, disparities in health care infrastructures, training opportunities, and available resources across countries should be taken into account when interpreting the results.

In conclusion, this cross-sectional survey provides insights into ECMO implementation from the perspective of health care professionals mostly from resource-constrained and transitional health care environments. The results highlight the necessity for standardized training programs, broader distribution of ECMO standards, and initiatives to overcome resource-related obstacles that restrict ECMO accessibility. As ECMO advances as a life-saving technology, especially following the COVID-19 pandemic, the establishment of comprehensive ECMO education and certification programs is essential for guaranteeing its safe and effective application in various health care environments.
