# Supplementary material for: Healthcare professionals’ views on training, standards, and resources for extracorporeal membrane oxygenation: a cross-sectional survey
Source: Croat Med J. 2025 Dec;66(6):419–28. doi: 10.3325/cmj.2025.66.419 (PMC12836024; doi:10.3325/cmj.2025.66.419)
Supplement: Supplementary Material 1 [file CroatMedJ_66_s006.pdf]

Supplementary Material 1

Quesionnaire

The current international survey is aimed to examine emergency medicine and allied specialists' knowledge and views on unmet needs in extracorporeal membrane oxygenation (ECMO) procedures in adults. The questionnaire will take about 10 – 15 minutes to complete. This questionnaire is designed based on related literature reviews, previous surveys, and relevant practice guidelines.

The questionnaire responses are expected to be based on responders' knowledge and experience of ECMO. By completing this survey questionnaire, respondents give their agreement to publicize their anonymized answers. The questionnaire is divided into 4 sections (basic knowledge of ECMO; perception of the main issues and unmet needs in ECMO; perspectives of ECMO procedures; socio-demographic information). Although there are some personal demographic and clinical experience questions about responders, all answers will be kept confidential and anonymized and will be used only for research purposes. Only questionnaires with complete answers to all points will be processed.

Thank you in advance for taking your time and sharing your professional knowledge to complete this questionnaire. We greatly appreciate the information you provide to advance the organization of ECMO locally and internationally.

If you have any questions related to this survey, please do not hesitate to contact the moderators of this study.

1. Are you familiar with the following definition of extracorporeal membrane oxygenation (ECMO) that was introduced in 1989 by the Medical Subject Headings (MeSH) of the National Library of Medicine of the US (https://www.ncbi.nlm.nih.gov/mesh/?term=extracorporeal+membrane+oxygenation): "Application of a life support system that circulates the blood through an oxygenating system, which may consist of a pump, a membrane oxygenator, and a heat exchanger. Examples of its use are to assist victims of SMOKE INHALATION INJURY; RESPIRATORY FAILURE; and CARDIAC FAILURE."
   Yes/No/Not sure
2. Are you familiar with guidelines of the Extracorporeal Life Support Organization (ELSO; https://www.elso.org/ecmo-resources/elso-ecmo-guidelines.aspx) on ECMO and related procedures?
   Yes/No/Not sure
3. Please list clinical conditions or diseases that should be managed by ECMO (Choose all that apply)
   Acute Respiratory Distress Syndrome/Other, please specify

4. Please indicate your level of agreement with the following statement on the use of ECMO.
   Strongly disagree/Disagree/Neutral/Agree/Strongly agree
   ECMO is effective and safe for treating critically-ill patients
5. Who should be involved in ECMO procedures to avoid adverse effects and increase survival of patients? (Choose all that apply)
   Cardiothoracic surgeon/Non-Cardiothoracic surgeon/Intensivist/Cardiologist/Perfusionist/Respiratory Therapist (Pulmonologist)/Certified ECMO Specialist/ECMO Nurse
6. Please mark your agreement with the following statement (Choose only one option)
   Only specialists with ECMO certification should be involved in performing ECMO.
   Strongly disagree/Disagree/Neutral/Agree/Strongly agree
7. Which of the following are indications for ECMO? (Choose all that apply)
   Postcardiotomy (surgical interventions on heart valves and septum)/Heart transplantation/Lung transplantation/Cardiogenic shock/Respiratory failure (Acute Respiratory Distress)/Cardiopulmonary failure/Intoxication/Trauma or drowning
8. When making decisions to initiate or discontinue ECMO, which of the following factors should be considered?
   Age/Comorbidities/Overall clinical benefits for patients/Survival chances/Patients' quality of life after ECMO/Duration and expenses related to ECMO procedures/Opinion of the patients' family members
9. Does your medical centre offer ECMO procedures?
   Yes/No/Not sure
10. On average, how many ECMO procedures does your medical centre offer per year?
    None/Less than 10/10-30/31-50/More than 50/Not sure
11. How long has your medical centre offered adult ECMO procedures?
    Less than 2 years/2-5 years/6-10 years/More than 10 years/Not sure
12. Which modes of ECMO are offered at your medical centre? (Choose all that apply)
    None/Venoarterial (VA)/Venoarterial plus additional venous drainage (VA+V)/Venovenous (VV)/VV using double lumen cannula (VVDL)/VVDL plus additional venous drainage (VVDL+V)
13. Which anticoagulant would you prefer for initiating ECMO, particularly in case of VA-ECMO procedures for adults? (Choose all that apply)

Heparin/Unfractionated heparin (UFH)/Argatroban/Bivalirudin/Warfarin/Dabigatran/Rivaroxaban

14. Which could be your strategy of antimicrobial drug therapy for ECMO procedures in adults? (Choose all that apply)
    Therapy based on characteristics of antimicrobials (protein binding property, molecular weight, hydrophilicity/lipophilicity)/No different from strategies applicable to therapies in critically-ill patients without ECMO/Not sure
15. Does your medical centre offer ECMO training?
    Yes/No/Not sure

16. What type of training/certification is required for performing ECMO procedures? Choose all that apply
    Residency certification in a relevant specialty (e.g., cardiac surgery, emergency medicine)/ECMO didactic course at a specialist centre with lectures, seminars, and provision of handouts/ECMO scenario-based simulation training/Certain number of supervised ECMO cannulations/Attendance of ECMO-related local and international congresses/conferences/symposiums
17. Please rate the following statements on a scale of 1 to 5
    (not important) 1 2 3 4 5 (extremely important).
    1/2/3/4/5/
    Didactic teaching by highly skilled ECMO specialists is important for effective ECMO education.
18. Please rate the following statements on a scale of 1 to 5
    (not important) 1 2 3 4 5 (extremely important).
    1/2/3/4/5/
    Simulation-based training is important for effective ECMO education.
19. Please rate the following statements on a scale of 1 to 5
    (not important) 1 2 3 4 5 (extremely important).
    1/2/3/4/5/
    Individual/institutional membership in ECMO-related associations such as the Extracorporeal Life support Organisation (ELSO) is important for gaining and maintaining skills in ECMO procedures.
20. Please rate the following statements on a scale of 1 to 5
    (not important) 1 2 3 4 5 (extremely important).
    1/2/3/4/5/
    Doing research in the field of ECMO is important for gaining and maintaining skills in ECMO.
21. Have you been involved in ECMO-related research/clinical trial/registry reporting/systematic or narrative review writing?
    Yes/No
22. What are perceived barriers to widespread use of ECMO in your medical centre/city/country? Choose all that apply
    High costs of ECMO procedures/Lack of resources or inaccessibility of ECMO devices/Limited number of ECMO trained doctors/Limited number of ECMO trained nurses/High mortality/Scarce scientific evidence to support ECMO use/Lack of adapted practice guidelines on ECMO procedures/Other, please specify
23. Have you managed critically-ill COVID-19 patients referred to ECMO? Yes/No
24. Based on your experience, to what extent, did the COVID-19 pandemic affect the organization of ECMO procedures in your healthcare setting?
    Not affected at all/Affected to some extent/Significantly affected
25. Based on your experience, which of the following has changed in the COVID-19 pandemic period? (Choose all that apply)

Increased referral of patients with COVID-19 to ECMO/Increased referral of non-COVID-19 patients to ECMO/Other, please specify

26. Please indicate your level of agreement with the following statement on the use of ECMO for critically-ill COVID-19 patients.
    Strongly disagree/Disagree/Neutral/Agree/Strongly agree
    ECMO is effective for management of critically-ill COVID-19 patients.
27. Please indicate your level of agreement with the following statement on the use of ECMO for critically-ill non-COVID-19 patients.
    Strongly disagree/Disagree/Neutral/Agree/Strongly agree
    ECMO is effective for management of critically-ill non-COVID-19 patients.
28. What would you recommend to improve the efficiency of ECMO procedures?
    Open-ended question
29. Your gender
    Female/Male/Not specified
30. Your age
31. Which of the following best indicates your specialty background? (Check all that apply)
    ECMO Specialist Physician/ ECMO Specialist Nurse/Intensivist/Anesthesiologist/Pulmonologist/Cardiologist/Cardiac Surgeon/Vascular Surgeon/Emergency Physician/Trauma Surgeon/Clinical Toxicologist/Perfusionist/Nurse/Other, please specify
32. Which country do you practise in?
33. Which of the following best describes your medical centre?
    Public hospital/Private hospital/University-affiliated hospital/Other, please specify
34. How long have you been practicing as a health professional?
    Less than 1 year/1-5 years/more than 5 years
35. How long have you been practicing in emergency medicine?
    Less than 1 year/1-5 years/more than 5 years
36. Does your healthcare setting have a specialized department/unit for ECMO?
    Yes/No
37. Location of your healthcare setting
    Urban/Rural
